# Serum antibodies to the HPV16 proteome as biomarkers for head and neck cancer

**DOI:** 10.1038/bjc.2011.171

**Published:** 2011-06-07

**Authors:** K S Anderson, J Wong, G D'Souza, A B Riemer, J Lorch, R Haddad, S I Pai, J Longtine, M McClean, J LaBaer, K T Kelsey, M Posner

**Affiliations:** 1 Cancer Vaccine Center, Dana-Farber Cancer Institute, Boston, MA 02115, USA; 2 Department of Medical Oncology, Dana-Farber Cancer Institute, Boston, MA 02115, USA; 3 Department of Epidemiology, Johns Hopkins Bloomberg School of Public Health, Baltimore, MD 21205, USA; 4 Department of Otolaryngology-Head and Neck Surgery, Johns Hopkins Medical Institutions, Baltimore, MD 21287, USA; 5 Department of Pathology, Brigham and Women's Hospital, Boston, MA 02115, USA; 6 Department of Environmental Health, Boston University School of Public Health, Boston, MA 02118, USA; 7 Center for Personalized Diagnostics, Biodesign Institute, Arizona State University, Tempe, AZ 85287, USA; 8 Bio Med Center for Environmental Health and Technology, Brown University, Providence, RI 02912, USA; 9 The Tisch Cancer Institute, Mount Sinai School of Medicine, New York City, NY 10029, USA

**Keywords:** autoantibodies, biomarker, Luminex, HPV, head and neck cancer

## Abstract

**Background:**

Human papillomavirus (HPV) type 16 is associated with oropharyngeal carcinomas (OPC). Antibodies (Abs) to HPV16 E6 and E7 oncoproteins have been detected in patient sera; however, Abs to other early HPV-derived proteins have not been well explored.

**Methods:**

Antibodies to the HPV16 proteome were quantified using a novel multiplexed bead assay, using C-terminal GST-fusion proteins captured onto Luminex beads. Sera were obtained from untreated patients with OPC (*N*=40), partners of patients with HPV16+ OPC (*N*=11), and healthy controls (*N*=50).

**Results:**

Oropharyngeal carcinomas patients with known virus-like capsid particle+ Abs had elevated serum Abs to HPV16 E1, E2, E4, E6, and E7, and L1 antibody levels, but not E5. The ratios of specific median fluorescence intensity to p21-GST compared with controls were E1: 50.7 *vs* 2.1; E4: 14.6 *vs* 1.3; E6: 11.3 *vs* 2.4; E7: 43.1 *vs* 2.6; and L1: 10.3 *vs* 2.6 (each *P*⩽0.01). In a validation cohort, HPV16 E1, E2, and E7 antibody levels were significantly elevated compared with healthy control samples (*P*⩽0.02) and partners of OPC patients (*P*⩽0.01).

**Conclusion:**

Patients with HPV16+ OPC have detectable Abs to E1, E2, and E7 proteins, which are potential biomarkers for HPV-associated OPC.

Approximately 20 million Americans are currently infected with human papillomavirus (HPV), and another 6.2 million people become newly infected each year (Dunne *et al*, 2007; Markowitz *et al*, 2009). Genital HPV is a common sexually transmitted disease, with prevalent infection in a quarter of all adult women (Dunne *et al*, 2007; Markowitz *et al*, 2009). Recently, HPV infection has been identified as an aetiologic cause of a rapidly increasing subset of oropharyngeal carcinomas (OPC) (Loning *et al*, 1985; Andl *et al*, 1998; Wilczynski *et al*, 1998; Mork *et al*, 2001; van Houten *et al*, 2001; Ringstrom *et al*, 2002). There are an estimated 82 962 HPV-associated OPC cases per year worldwide. Oropharyngeal carcinomas is increasing in incidence in North America and HPV DNA is now found to be present in 50–70% of OPC cases in North America and to a lesser extent in Europe. Contrary to other HPV-related malignancies, HPV16 type accounts for 85–90% of HPV-associated cases of OPC (D'Souza *et al*, 2007; Marur *et al*, 2010). Human papillomavirus type 16-associated OPC has a significantly improved clinical outcome and responsiveness to therapy (Gillison *et al*, 2000; Schwartz *et al*, 2001; Furniss *et al*, 2007; Chung and Gillison, 2009; Ang *et al*, 2010; Posner *et al*, 2011).

Initial HPV infection induces both systemic and local humoral immune responses (Dillner *et al*, 1994; Kirnbauer *et al*, 1994; Schwartz *et al*, 1998). Serum antibodies (Abs) to L1 capsid protein are induced in 50–70% of infected patients months after HPV infection and are detectable for years after clearance of the infection, thus capsid Abs represent a history of lifetime HPV exposure (Wang *et al*, 2004; Frazer, 2009). Detection of serum Abs to HPV16 E6 and E7 are induced in a subset of patients with invasive cancers (Zumbach *et al*, 2000; Herrero *et al*, 2003; Smith *et al*, 2007a; Reuschenbach *et al*, 2008). The frequency of virus-like capsid particle (VLP), E6, and E7 seropositivity is directly associated with the presence of HPV DNA in the tumour (Rosales *et al*, 2001; Kreimer *et al*, 2005). Seropositivity for HPV16 E6 or E7 is strongly associated with the odds of OPC (64% of cases; OR: 58) (D'Souza *et al*, 2007), and predicts an improved prognosis (Smith *et al*, 2008a). Humoral immunity to other HPV early gene products has not been well evaluated, and the relationship of early gene Abs to the pathogenesis and prognosis of OPC and other HPV-related cancers has not been established. As the incidence of HPV-related OPC is predicted to increase over the next three decades, a broader look at the immune responses to the entire HPV proteome may yield sensitive biomarkers for early identification of at-risk populations, prediction of biologic behaviour and therapeutic outcome, and detection of second HPV malignancies.

We have developed rapid, programmable, multiplexed serologic assays for the detection of antigen-specific Abs in human sera (Ramachandran *et al*, 2004, 2008a, 2008b; Anderson and LaBaer, 2005; Anderson *et al*, 2008). This approach uses mammalian *in vitro* expression of cDNA's encoding protein antigens, followed by the capture of antigens using anti-tag Abs, and subsequent binding and multiplexed detection of Abs from patient sera. We have successfully expressed and captured over 15 000 different human, viral, and bacterial proteins for monitoring humoral immunity, and have adapted this approach for bead-based (Luminex) arrays (Wong *et al*, 2009) for high-throughput serologic screening. Because this approach is based on cDNA rather than on purified proteins, it allows for greater flexibility and economy in antigen selection and for epitope mapping.

To evaluate immune responses to HPV proteins as potential biomarkers for early detection of OPC, we developed a programmable Luminex bead array ELISA for the detection of serum Abs to the HPV16 proteome from patients with HPV+ and HPV− OPC, partners, and controls. In parallel, we have used *in situ* hybridisation for the characterisation of HPV16 content in tumours. The marked heterogeneity of immune responses in the uniform patient population suggests that there are fundamental biologic differences in host/viral biology that may impact clinical outcome.

## Materials and methods

### Study populations

#### Brown University case/control training dataset

Human papillomavirus serology was performed using retrospectively identified sera from 20 head and neck cancer patients (‘training set’) collected at Brown University that were known to be positive for HPV16 (*N*=10) or HPV18 (*N*=10) Abs defined by the competitive Luminex immunoassay (cLIA; VLP Ab+) developed by Merck (West Point, PA, USA) (Smith *et al*, 2008c). Control sera (*N*=20) were obtained from Brown University and were age (±5 years), residence, and gender matched to cases and processed simultaneously. Serum samples were collected using a standardised sample collection protocol and stored at −80 °C until use.

#### Validation (Dana-Farber Cancer Institute/Johns Hopkins University) case/control data set

To confirm the findings from the training set, serum from 30 pre-therapy OPC cases and 30 controls, collected as part of a larger research study, were evaluated. This included blood from OPC patients at Dana-Farber Cancer Institute (DFCI, *N*=17) and Johns Hopkins University (JHU, *N*=13) as well as healthy anonymous blood donors (*N*=30) and partners of OPC patients (*N*=11). Of these samples, 55 were sera and 16 were plasma (6 of these were obtained at the time of leukopheresis).

#### HPV16+ healthy control (CDC) dataset

We also evaluated serum from women with documented cervical infection with HPV16. The sera were obtained from an NCI Early Detection Research Network biorepository collected from women attending colposcopy clinics (Rajeevan *et al*, 2005). A total of 20 serum samples were identified from subjects that were positive for HPV16 DNA in exfoliated cervical cells using the Roche prototype line blot assay (reagents provided as a gift from Roche Molecular Systems, Inc., Pleasanton, CA, USA). All subjects had no cervical disease (CIN 0) at the time of colposcopy. Written informed consent was obtained from all subjects under institutional review board approval.

### Gene cloning

Human papillomavirus types 16 and 18 genes E1, E2, E4, E5, E6, E7, L1, and L2 were obtained by nested PCR. As HPV16 E2 expressed poorly, it was fragmented into N- and C-terminal halves. An initial PCR was done with gene-specific primers (Supplementary Table 1) from HPV16 and HPV18 purified plasmid DNA (American Type Culture Collection, Manassas, VA, USA). Primer extension PCR was used to add attB sites for recombination cloning. The att PCR products were inserted into the pDONR221 vector according to manufacturer's recommendations using BP Clonase (Invitrogen, Carlsbad, CA, USA) and were converted to the pANT7_GST vector (Ramachandran *et al*, 2004) with LR recombinase (Invitrogen). DNA was prepared with Nucleobond Xtra Maxi (Macherey-Nagel Inc., Bethlehem, PA, USA) and sequence confirmed.

### Bead array ELISA

SeroMAP carboxylated microspheres (Luminex Corporation, Austin, TX, USA) from regions #35–44 were coupled at a ratio of 5 *μ*g anti-GST antisera to 1 million beads and the coupling confirmed as described in Wong *et al* (2009). Each HPV gene was expressed as GST-fusion proteins using a single batch of T7 reticulocyte lysate (Promega Corporation, Madison, WI, USA) per manufacturer's recommendations with 500 ng DNA. Vector and p21-GST were also expressed as negative controls. The *in vitro* transcription/translation (IVTT) products were each captured onto 2000 microspheres at 40 microspheres per *μ*l in PBS-1% BSA. Protein-bound microspheres were pooled together, then re-aliquoted to a 96-well filter plate (Millipore Corporation, Billerica, MA, USA). Microspheres were blocked with 10% each of normal sera from mouse, rabbit, goat, and rat; 0.5% polyvinyl alcohol (Sigma-Aldrich, St Louis, MO, USA); 0.8% polyvinylpyrrolidone (Sigma-Aldrich); and 2.5% Chemicon (Millipore) in PBS-1% BSA. Test sera were diluted 1 : 80 in blocking buffer and incubated with the washed beads overnight at 4 °C while shaking. Biotin-conjugated goat anti-human IgG antibody (4 *μ*g ml^–1^, Jackson ImmunoResearch Laboratories, Inc., West Grove, PA, USA) and streptavidin-R-PE (4 *μ*g ml^–1^, Molecular Probes, Inc., Eugene, OR, USA) were added. To control for non-specific and GST-specific autoantibody background, the ratio of median fluorescence intensity (MFI) for individual HPV-specific Abs to the MFI for the control p21-GST antigen was measured. To determine protein expression, GST-protein tags were detected with anti-GST monoclonal Ab (Cell Signaling Technology, Danvers, MA, USA) and PE-conjugated goat anti-mouse IgG (Jackson ImmunoResearch Laboratories).

### Tumour HPV DNA detection by PCR

DNA was isolated from paraffin-embedded tumour tissue (QIAamp DNA mini kit, QIAGEN, Valencia, CA, USA). Polymerase chain reaction was performed using a procedure developed by Access Genetics (Minneapolis, MN, USA), which amplifies the HPV L1 domain with degenerate forward and reverse primers (Agoston *et al*, 2010). Each 50 μl reaction contained 1.25 units of *Taq* polymerase (Fisher Scientific, Pittsburgh, PA, USA), 10 mmol l^–1^ Tris–HCL, pH 9.0, 50 mmol l^–1^ KCl,4.6 mmol l^–1^ MgCl_2_, 0.02 mmol l^–1^ of each deoxyribonucleotide triphosphate, 11 pmol of each primer, and 100–500 ng of DNA. The reactions were cycled at 95 °C for 2 min, followed by 20 s each of 95 °C then 55^ ^°C and 30 s at 72 °C. The final cycle was 72 °C for 5 min and held at 15 °C until analysed. As a control for DNA quality, the DNA was separately amplified with primers to the *β*-globin gene (Forward: AGAATGGTGCAAAGAGGCATGA, Reverse:GCATCAGTGTGGAAGTCTCAGG; 503 bp product). The DNA samples that were positive for HPV were genotyped by restriction endonuclease fragment analysis. The HPV PCR products were digested in three separate reactions using PstI, Rsa I, and Hae III. All PCR products were resolved on 1 mm, 5% acrylamide gels, stained with ethidium bromide, and visualised by transillumination. The pattern of restriction fragment-length polymorphism of each sample was compared with the Access Genetics database to determine the HPV genotype (Ralston Howe *et al*, 2009).

### Statistical analysis

Serum HPV Ab levels were measured as MFI using the Luminex200 IS 2.3 software. Fifty events were counted for each bead region. To establish ELISA cutoff values, the training set control sera (*N*=20) and the validation control sera (*N*=30) were used. An MFI ratio > (the average + 3 s.d.) of all control samples was designated positive. These levels were E1: 5.4, NE2: 8.5, CE2: 6.8, E4: 2.3, E5: 4.2, E6: 9.0, E7: 6.6, L1: 9.5, and L2: 8.0. To determine the variability of serum detection for each HPV antigen, test serum from a single OPC patient (tumour HPV16+ confirmed by PCR) was assayed in duplicate on four separate days for each of the HPV16 antigens. Intra-assay and inter-assay variability was determined by calculating the coefficient of variation (CV, (s.d./mean) × 100). Heat maps and cluster analysis were done using MultiExperiment Viewer software (Saeed *et al*, 2003).

## Results

### HPV16 bead array ELISA

**Figure 1:**
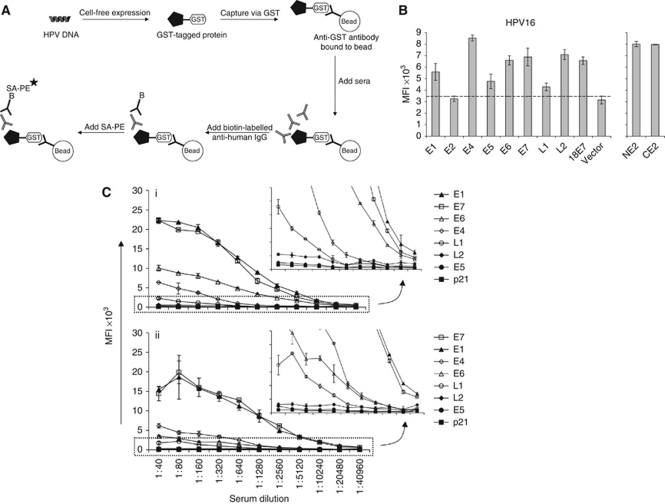
HPV bead array ELISA. (**A**) Schematic of bead array ELISA for the detection of HPV Abs. Individual HPV cDNA's encoding full-length HPV antigens are expressed as C-terminal GST-tagged proteins using reticulocyte lysate. The expressed protein is then captured onto Luminex microspheres via anti-GST antibody that is coupled onto the beads. For IgG detection from sera, beads tagged with HPV antigens are mixed and added to human sera. Bound human IgG is detected with biotin-anti-IgG and streptavidin-PE. (**B**) GST expression of HPV16 gene products and HPV18 E7. GST expression of N- and C-terminal HPV16 E2 fragments is shown in the right panel. GST protein on the beads was measured by addition of anti-GST-PE antibody as mean fluorescence index (MFI). Background protein expression (vector mean plus 10%) is indicated by the dotted line. (**C**) Antibodies to HPV16 early gene proteins are detected at >1 : 10 000 dilution in two OPC patient sera. Patient sera were diluted two-fold from 1 : 40 to 1 : 40 960, and IgG Abs to HPV-derived proteins and p21-GST control protein are shown in order of peak MFI signal intensity. Inserts: zoom-in views of (i) and (ii), respectively.

A schematic of the HPV16 bead array ELISA for the detection of Abs in patient sera to the HPV16 antigens is shown in Figure 1A. The GST expression of all HPV16 (E1, NE2, CE2, E4, E5, E6, E7, L1, and L2), as well as HPV18 E7-fusion proteins are shown in Figure 1B. Protein expression was defined as over 10% greater than the average vector control signal, indicated by the dotted line (Figure 1B), and is consistent with observed protein expression levels from >70 IVTT-expressed antigens on bead arrays (Wong *et al*, 2009) and >7000 antigens on slide-based microarrays (Ramachandran *et al*, 2008b). The cutoff value was 3458 MFI with signals ranging between 2997 and 8525 MFI (mean=5870). Human papillomavirus type 16 E2 was considered a non-expressor and fragmented into N- and C-terminal halves with strong expression of both fusion proteins (Figure 1B, right).

In prior studies, we determined that IVTT-based protein display ELISAs demonstrate comparable sensitivities and limits of detection as recombinant protein ELISA for the detection of both p53- and EBV-specific Abs (Anderson *et al*, 2008; Ramachandran *et al*, 2008a) and can multiplex eight antigen-specific Abs with long-term stability and without loss of sensitivity (Wong *et al*, 2009). The reproducibility of Abs to all HPV16 gene products was measured in duplicate using serum from an HPV16+ OPC patient four times over a 1-week span. Intra-assay CV's ranged from 0.19 to 22.4% and inter-assay CV's ranged from 7.6 to 21.3% (Supplementary Table 2). Positive control sera for E1, E2, and E7 Abs were included with every assay, with 100% accuracy measured for 8–16 assays over 12 months using the cutoff values established (see Materials and Methods).

### High-titre Abs to HPV16 antigens are detected in representative OPC patient sera

IgG Abs specific for HPV-derived proteins were detected in two representative serially diluted OPC patient sera (Figure 1C). For both sera, Abs to E1 and E7 were detected with the highest signals, with peak signal detection at a serum dilution of 1 : 80, which has also been observed with the highly immunogenic EBNA-1 and p53 antigens using the bead array ELISA (Wong *et al*, 2009). Half-maximal signal occurred at a serum dilution of 1 : 640, and E1- and E7-specific Abs could be detected at >1 : 10 000 dilution. Antibodies to L2, E5, and the negative control p21 antigen were not detected, with raw signals between 28.5 and 548 MFI. Median fluorescence intensity of p21-GST did not differ between case and control samples in the training and validation sets (*P*=0.524). For all subsequent studies, the MFI ratio of specific HPV Ab to p21-GST was used to control for non-specific protein binding.

### Serum Abs to HPV16 genes in OPC cases compared with controls: training set

We initially selected a retrospective training set of sera from patients known to have Abs to HPV16 VLP or HPV18 VLP by the highly sensitive Merck cLIA assay, which were predicted to have higher levels of Abs to HPV-derived early genes. The characteristics of patients with HPV-associated OPC and age- and gender-matched controls (training set) are shown in Table 1. History of alcohol and tobacco use was similar among cases and controls. In all, 18 of 20 of the cases had OPC cancer; 2 had laryngeal cancer; and 16 of 20 had stage III/IV cancers. The status of HPV viral DNA in the tumour for the training set cases is not known.

**Figure 2:**
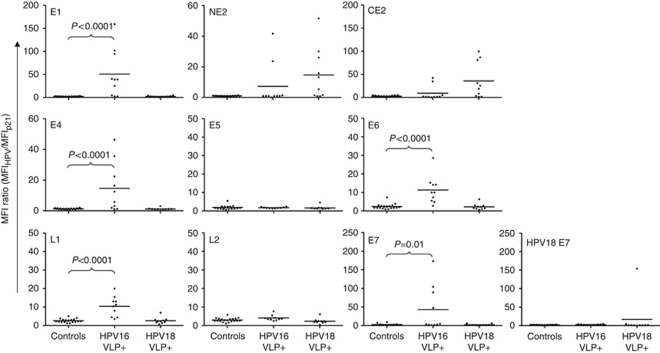
Specific detection of multiple early gene Abs in 10 patients with HPV16 VLP Abs compared with 20 controls. HPV16 proteins, as well as HPV18 E7 and p21 proteins, were expressed and captured on Luminex beads, and the MFI ratio (MFI (HPV)/MFI (p21-GST)) of IgG detected in sera is shown. Training set serum IgG responses were measured in age- and sex-matched healthy controls (‘Controls’), patients with HPV16 VLP Abs (‘HPV16 VLP+’), and patients with HPV18 VLP Abs (‘HPV18 VLP+’). HPV16-specific Abs to E1, E4, E6, E7, and L1 proteins (but not E5 and L2) are detected in HPV16 VLP Ab+ patients compared with controls or patients with HPV18 VLP Abs. Top middle and top right: HPV16 NE2 and CE2 Abs are also detected in cases with HPV18 VLP Abs. Bottom right middle and far right: HPV16 E7 Abs do not cross-react with HPV18 E7.

Among the training set of 20 head and neck cancer sera and 20 controls, HPV16-specific Abs to E1, E4, E6, E7, L1, and L2 were significantly more common among OPC cases with HPV16 VLP Abs than in healthy controls (each *P*⩽0.05) (Figure 2; Table 2). There was no specific detection of E5 Abs in cases compared with controls. To control for exposure to other HPV types, 10 OPC patients with known HPV18 VLP Abs, but not HPV16 VLP Abs in the sera, were also evaluated. The status of HPV viral DNA in the tumours was not known (i.e., these were not known HPV18+ tumours); these patients had evidence of prior infection with HPV18 based only on VLP Abs. This cohort had no evidence of HPV16-specific Abs to E1, E4, E6, or E7. A subset of patients with HPV18 VLP had Abs to NE2 and CE2, suggesting that there is viral type-specific cross-reactivity of this antigen.

To determine if there was cross-reactive antigenicity between viral types, Abs to HPV16E7 and HPV18E7 in patient sera were compared. Patients who had detectable Abs to HPV16E7 did not have detectable Abs to HPV18E7 (Figure 2). One patient with Abs to HPV18 VLP also had detectable Abs to HPV18E7. Only 6 out of 10 HPV VLP Ab+ patients also had detectable L1 Abs by bead array ELISA, likely representing display of different antigenic structures detected in the two assays, or a lower sensitivity of the bead array ELISA for the detection of L1 Abs. Of the four L1 Ab-negative cases, there was no significant difference in background p21 MFI compared with the L1 Ab+ cases (*P*=0.914). No correlation between HPV16L1 bead array signal intensities and VLP titre were observed (*R*=0.638), likely related to structural differences in epitope display, which has been observed for L1-specific epitopes (Vonka *et al*, 1998).

### Serum Abs to HPV16 genes in OPC cases compared with controls: validation set

**Figure 3:**
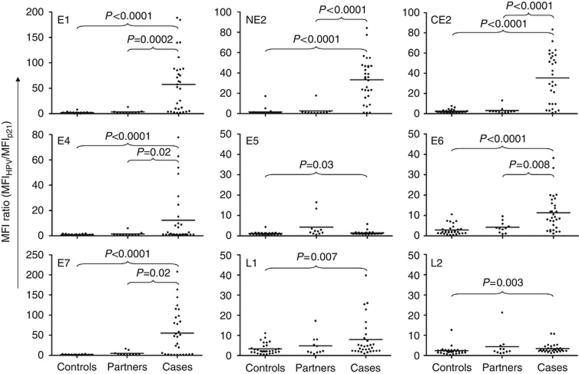
Specific detection of multiple early gene Abs in untreated OPC patients, but not partners. The MFI ratio of HPV16 proteins to p21 protein detected in sera is shown. The validation set serum IgG responses were measured in an independent set of healthy controls (‘Controls’, *N*=30), OPC patients (‘Cases’, *N*=30), and partners of OPC patients (*N*=11). HPV16-specific Abs to E1, E2, E4, E6, and E7, and L1 proteins are specifically detected in patients compared with controls.

As the training set sera were specifically selected for head and neck cancer patients with L1-specific Abs, we evaluated OPC patients at DFCI and JHU with unknown serum HPV L1 status, compared with controls. The clinical characteristics of the OPC patients are shown in Table 3. Human papillomavirus type 16 E1, NE2, CE2, E4, E6, E7, and L1-specific Abs were significantly more common among OPC patient sera compared with healthy controls (E1, NE2, CE2, E4, E6, E7, and L1, each *P*<0.007) (Figure 3; Table 4). There was no specific detection of E5 or L2 Abs in cases. In this unselected cohort, the detection of L1-specific Abs was only 23% (7 out of 30) compared with healthy controls (1 out of 30, 3%).

The HPV Abs most strongly associated with OPC were HPV16E1 (22 out of 30 (73%) of cases *vs* 1 out of 30 controls), NE2 (25 out of 30 (83%) of cases *vs* 1 out of 30 controls), CE2 (24 out of 30 (80%) of cases *vs* 1 out of 30 controls), and HPV16E7 (19 out of 30 (63%) of cases *vs* 0 out of 30 controls), *P*<0.001. Compared with E1, E2, and E7 Abs, fewer patients had E4 Abs (13 out of 30 (43%) *vs* 0 out of 30 controls) or E6 Abs (15 out of 30 (50%) *vs* 1 out of 30 controls). All 16 patients with known HPV16+ tumours by PCR had detectable E1, E2, or E7 Abs. Four of the OPC patients had HPV16-negative tumours by PCR; of these, two had detectable Abs to both E1 and E7. This may reflect cross-reactivity with other HPV types, limitations of the HPV PCR assay, or represent HPV16 infection at other sites. As this cohort included a mix of sera and plasma, a direct comparison of the subset of serum cases and controls was performed, with the specificity of detection of HPV16 E1, E2, E6, and E7 Abs maintained (*P*<0.002).

Given the high proportion of adults (>70%) having ever been infected with high-risk (HR) HPV in most populations (Markowitz *et al*, 2009), we anticipate that the majority of our healthy controls have been exposed to HPV at some point. To evaluate the correlation between Abs to HPV16 early (E) proteins and current HPV16 infection, we identified 20 healthy controls with known HPV16 DNA present in cervical exfoliated cells, but with normal cervical colposcopies (CIN 0; no evidence of cervical disease). None of these controls had evidence of HPV16 Abs to E1, E2, E6, or E7 (data not shown), suggesting that the presence of early gene Abs in cases is not simply a marker of active HPV16 infection.

### HPV16 early gene Abs in partners of HPV16+ OPC patients

The recent identification of HPV-associated OPC has raised the question of whether healthy partners of these patients, who are chronically exposed to oncogenic HPV from their partners with cancer, develop protective immunity to HPV or are at risk for the subsequent development of HPV-associated malignancies. Antibodies to HPV16 antigens were measured in sera from partners of patients with OPC (*N*=11, 7 partners known to have HPV16+ tumours; Figure 3). Low-level Abs to each gene product were detected in sera of one or two partners of patients with known HPV16+ tumours, with one partner of a patient with HPV16 status unknown having Abs to five different gene products.

### HPV16 antibody hierarchical clustering

**Figure 4:**
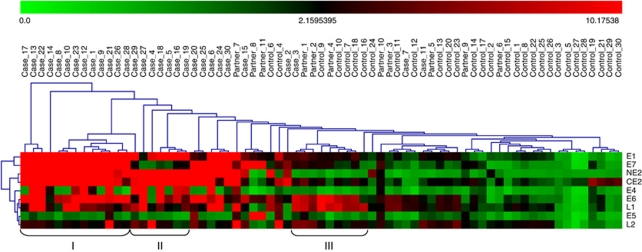
Unsupervised hierarchical clustering of HPV16-specific Abs of validation set patient, partner, and control sera. There is a subset of patients (group I) with multiple HPV-specific Abs, including E1, E2, E6, and E7. Another subset (group II) has E1 and E2, but low or absent E7- and/or E6-specific Abs. There is a subset (group III) of healthy controls with increased (but weak) levels of E6 and L1 Abs.

To determine whether antibody responses to specific HPV early genes are concordant (are detected in the same patient sera), the HPV16 antibody responses in the validation set detected by bead array ELISA were grouped by unsupervised hierarchical clustering analysis (Figure 4). Distinct patterns of Ab reactivity were observed. Pattern I were cases with a strong detection of E1, E2, E7, and E6 Abs. Pattern II consisted of cases with E1 and E2 Abs, but not E6 and/or E7 Abs. Pattern III were controls and partners with weak E6 and L1 Abs, suggesting that there is low-level antibody immunity in this population that may be protective.

## Discussion

It is estimated that 75–80% of sexually active persons in the United States will be infected with HR HPV in their lifetime (Dunne *et al*, 2007). Although effective HPV-prevention vaccines targeting the L1 capsid protein are currently available, the majority of people over the age of 21 has already been infected with HR HPV, either genitally or orally, and may, therefore, remain at risk of OPC. Clinical biomarkers such as HPV-specific Abs to identify HR individuals infected with oncogenic HPV types could have significant clinical impact and long-term health implications.

The detection of serologic immunity to HPV infections has been hampered by the lack of standardised assays for antibody detection to the targets of natural immunity (Ferguson *et al*, 2009). Antibodies to capsid proteins have been measured by ELISA to conformationally intact VLPs, directly conjugated (Opalka *et al*, 2003; Dias *et al*, 2005) or indirectly conjugated via heparin (Faust *et al*, 2010) to Luminex microspheres for multiplexed detection of Abs to different HPV capsid types. To detect Abs to HPV16 E6 and E7, *Escherichia coli*-derived proteins have been detected by ELISA (Di Bonito *et al*, 2006) or adhered to Luminex beads (Waterboer *et al*, 2005; Reuschenbach *et al*, 2008) for multiplexed detection from single serum samples. Protein arrays displaying printed *E. coli*-derived HPV proteomes have recently been used to identify E7-specific Abs in cervical cancers (Luevano *et al*, 2010). However, expression and purification of proteins in non-mammalian systems may result in altered antigenic structure of viral proteins.

We have developed a novel assay for the detection of HPV16-specific IgG Abs in human sera. We demonstrate that Abs to multiple HPV16-derived early proteins, in particular E1, E2, E6, and E7, are present in pre-treatment sera of patients with incident HPV16+ OPC, but not in healthy controls or in a limited set of partners of patients with HPV16+ OPC cancer. Human papillomavirus type 16 E1 and E2 Abs, in particular, were detected in a small subset of patients who were negative for HPV16 E6 and E7 Abs, suggesting that inclusion of these serologic biomarkers in screening with E6 and E7 Abs may increase sensitivity and specificity of detection of infected patients. Human papillomavirus-specific Abs to E1, E2, and E7 were associated with the detection of HPV16 DNA within tumours in this small group and will need to be verified in larger cohorts. Antibodies to E2, in particular, may cross-react with other HPV types. Assays for the presence of HPV16 DNA in archived tumour samples has been evolving; for these studies, the assay was performed by experienced laboratories across the study sites and was performed within the past 12 months.

Of interest, these studies demonstrate that there is significant heterogeneity in the serologic response to HPV, even among patients with HPV16 detected in the tumour. We have not identified clinicopathologic criteria that are associated with the presence of patterns of HPV16-specific early gene Abs in this preliminary study, although male gender, younger age, and tumour burden have been associated with increased E6/E7 seropositivity (Smith *et al*, 2008b, 2010). Other studies have detected Abs to E6 or E7 in 65–70% of HPV16+ OPC patients, and are associated with improved clinical outcome (Smith *et al*, 2008a, 2010). In our findings, immune responses to the E6 antigen in patients are less frequent in OPC cases (50%) than reported and of much lower titre compared with Abs to E1/E2/E7. This may reflect technical differences in antigenic display between our serologic assay and the assay developed in the Pawlita laboratory (Waterboer *et al*, 2005); the assays have not been directly compared. While we do not observe cross-reactivity between HPV16 E7 and HPV18 E7 (the two dominant HPV types in OPC cancer), further studies correlating serology with the viral type detected in tissue will be needed.

In cervical cancer, Abs to 16E6 and 16E7 develop late in the course of disease and have been shown to correlate with disease response (Ravaggi *et al*, 2006). In an extensive Scandinavian study of pre-diagnostic sera collected 0.5–20 years before diagnosis of cervical cancer (*N*=178), 16E6 and 16E7-specific IgG were detected in 7% of cases with a relative risk (odds ratio) of 2.7, found 0.5–5 years before diagnosis (Lehtinen *et al*, 2003). This suggests that serology of the early HPV genes, in particular, may be detected prior to the development of overt malignancy. The utility of HPV Abs to predict increased risk of OPC have not been well evaluated.

The previously unreported E1- and E2-specific Abs in our cohort is striking because of both the high titre and high frequency in OPC patient sera, which improves the sensitivity of detection from 70% to 100% of HPV16+ OPC when combined with E6 and/or E7 Abs. Early studies demonstrated low immunoreactivity to an E1-derived peptide in cervical cancer sera (Dillner *et al*, 1995) and weak E1 Abs were identified in cervical cancer (Luevano *et al*, 2010); to our knowledge, Abs to E1 or E2 have not otherwise been described in cervical cancer nor in OPC. E1 proteins are essential origin recognition proteins for viral replication, binding to specific DNA elements and assembling into hexameric helicases (Wilson *et al*, 2002). Integration of HPV into the host genome results in disruption of E1/E2 expression and loss of repression of E6/E7 (Chung and Gillison, 2009). As a result, E1- and E2-specific Abs may develop prior to Abs to E6/E7 and might prove useful for early identification of risk.

Human papillomavirus antibody signatures may provide a rapid, convenient and inexpensive screening method for assessing the HPV status of suspected or known malignancy. Human papillomavirus+ OPC is a strong, independent factor for improved prognosis (Gillison *et al*, 2000; Schwartz *et al*, 2001; Furniss *et al*, 2007; Chung and Gillison, 2009; Ang *et al*, 2010), and HPV status is increasingly being incorporated into clinical trial stratification. However, current diagnostic studies for viral typing are cumbersome, expensive, delayed, and require tumour biopsies. Detection of viral load using oral swabs and rinses is in development (Summersgill *et al*, 2001; Smith *et al*, 2000, 2007b; Agrawal *et al*, 2008). It is not yet known whether HPV antibody signatures will have screening utility and/or have prognostic or biologic correlates for clinical outcome, monitoring therapy, or identifying early recurrence. In our uniform cohort of HPV16+ OPC patients, there is considerable variability in both the intensity and the specificity of the HPV Ab signatures, particularly for the early genes, suggesting that these biomarkers may identify clinically relevant subsets of patients. With the increasing number of cases of HPV-related oropharynx cancers occurring in North America and Europe, HPV cancer risk and prevention strategies will become an important part of routine medical care.

## Figures and Tables

**Table 1:** Characteristics at diagnosis of patients in the training set

	**Cases (*N*=20)**				
	**HPV16 VLP+^b^**	**HPV18 VLP+^b^**	**Controls**	***P*-value^a^**	
	***N*=10**	***N*=10**	***N*=20**	**HPV16 VLP+**	**HPV18 VLP+**
	***N* (%)**	***N* (%)**	***N* (%)**	***vs* controls**	***vs* controls**
*Age*					
47–56	5 (50)	3 (30)	8 (40)		
57–68	2 (20)	4 (40)	6 (30)		
69–84	3 (30)	3 (30)	6 (30)	0.82	0.83
					
*Sex*					
Female	1 (10)	5 (50)	6 (30)		
Male	9 (90)	5 (50)	14 (70)	0.22	0.28
					
*Ever smoked cigarettes?*					
Yes	8 (80)	7 (70)	12 (60)		
No	2 (20)	3 (30)	8 (40)	0.27	0.59
					
*Number of usual drinks per week*					
<4	2 (20)	1 (10)	7 (35)		
>4	8 (80)	9 (90)	13 (65)	0.40	0.14
					
*Stage*					
III	2 (20)	1 (10)			
IV	7 (70)	6 (60)			
Unknown	1 (10)	3 (30)			
					
*Tumour sub-site*					
Oral	1 (10)	3 (30)			
Pharynx	8 (80)	6 (60)			
Larynx	1 (10)	1 (10)			

Abbreviations: Abs=antibodies; cLIA=competitive Luminex immunoassay; HPV=human papillomavirus; VLP=virus-like capsid particle.

a *χ*^2^-test.

b Abs to HPV16 or 18 VLP by cLIA.

**Table 2:** Serum HPV antibody levels in patients with oropharyngeal cancer compared with non-cancer controls (training set)

**Antibodies**	**MFI ratio^a^**					
	**HPV16 VLP+ OPC cases (*N*=10)**		**HPV18 VLP+ OPC cases (*N*=10)**		**Controls (*N*=20)**	
**HPV16**	**Mean**	**(Range)**	**Mean**	**(Range)**	**Mean**	**(Range)**
**E1**	50.7^b^	(2.0–159)	2.2	(0.2–4.8)	2.1	(1.1–3.3)
NE2	7.2	(0.1–42)	14.7^c^	(0.8–52)	1	(0.4–1.4)
CE2	9.2	(0.3–42)	35.8^c^	(1.4–100)	2.7	(0.6–5.0)
**E4**	14.6^b^	(1.2–46)	1.2	(0.1–3.1)	1.3	(0.6–2.1)
E5	1.8	(1.3–2.5)	1.6	(0.1–4.6)	1.9	(0.7–5.5)
**E6**	11.3^b^	(2.8–29)	2.2	(0.6–6.3)	2.4	(0.9–7.3)
E7	43.1^c^	(1.4–173)	2.3	(0.2–6.1)	2.6	(1.0–9.6)
**L1**	10.3^b^	(3.5–20)	2.6	(0.7–6.9)	2.6	(1.2–5.1)
L2	4.2^c^	(2.6–7.7)	2.1^c^	(0.4–6.1)	3	(1.2–5.8)
						
*HPV18*						
E7	2.5	(1.4–4.3)	16.7	(0.3–154)	1.8	(0.5–2.8)

Abbreviations: HPV=human papillomavirus; MFI=median fluorescence intensity; OPC=oropharyngeal carcinomas; VLP=virus-like capsid particle.

a MFI ratio of HPV-GST antigen/p21-GST.

b Compared with controls, *P*<0.001 (bold).

c Compared with controls, 0.001⩽*P*⩽0.05 unpaired Wilcoxon's test.

**Table 3:** Characteristics at diagnosis of participants in the validation set

			**HPV16 E1 Ab^a^,b**			**HPV16 E7 Ab^a^,b**		
	**Cases**	**Partners**	**Positive**	**Negative**		**Positive**	**Negative**	
	***N*=30**	***N*=11**	***N*=22**	***N*=8**		***N*=19**	***N*=11**	
	***N* (%)**	***N* (%)**	***N* (%)**	***N* (%)**	***P*-value^c^**	***N* (%)**	***N* (%)**	***P*-value^c^**
*Age*								
39–46	3 (10)	3 (27.3)	2 (9.1)	1 (12.5)		2 (10.5)	1 (9.1)	
47–56	13 (43.3)	8 (72.7)	9 (40.9)	4 (50)		7 (36.8)	6 (54.5)	
57–68	12 (40)	0 (0)	10 (45.5)	2 (25)		8 (42.1)	4 (36.4)	
69–84	2 (6.7)	0 (0)	1 (4.5)	1 (12.5)	0.72	2 (10.5)	0 (0)	0.63
								
*Sex*								
Female	1 (3.3)	11 (100)	1 (4.5)	0 (0)		1 (5.3)	0 (0)	
Male	29 (96.7)	0 (0)	21 (95.5)	8 (100)	0.54	18 (94.7)	11 (100)	0.44
								
*Stage*								
II	3 (10)		1 (4.5)	2 (25)		2 (10.5)	1 (9.1)	
III	5 (16.7)		2 (9.1)	3 (37.5)		3 (15.8)	2 (18.2)	
IV	21 (70)		18 (81.8)	3 (37.5)	0.04	13 (68.4)	8 (72.7)	0.98
Unknown	1 (3.3)		1 (4.5)	0 (0)		1 (5.3)	0 (0)	
								
*Tumor sub-site*								
Pharynx	25 (83.3)		19 (86.4)	6 (75)		17 (89.5)	8 (72.7)	
Larynx	1 (3.3)		0 (0)	1 (12.5)	0.09	0 (0)	1 (9.1)	0.16
Unknown	4 (13.3)		3 (13.6)	1 (12.5)		2 (10.5)	2 (18.2)	
								
*HPV16 tumor*								
Yes	17 (56.7)		14 (63.6)	3 (37.5)		11 (57.9)	6 (54.5)	
No	4 (13.3)		3 (13.6)	1 (12.5)	0.74	2 (10.5)	2 (18.2)	0.59
Unknown	9 (30)		5 (22.7)	4 (50)		6 (31.6)	3 (27.3)	

Abbreviations: Ab=antibody; ELISA=enzyme-linked immunosorbent assay; HPV=human papillomavirus.

a Ab positive or negative by bead array ELISA.

b Cutoff = average + 3 s.d. of normals for each antigen.

c *χ*^2^-test.

**Table 4:** Serum HPV antibody levels in patients with oropharyngeal cancer compared with non-cancer controls (validation set)

	**Partners *N*=11**				**OPC cases** ***N***=**30**				**Controls *N*=30**			
**HPV16 Antibodies**	**MFI ratio^a^**	**(Range)**	**#Ab+**	**(%)**	**MFI ratio^a^**	**(Range)**	**#Ab+**	**(%)**	**MFI ratio^a^**	**(Range)**	**#Ab+**	**(%)**
**E1**	3.6	(1.5–13.2)	1	(9)	57.5^b^,c	(1.7–188.7)	22	(73)	1.9	(0.3–8.1)	1	(3)
**NE2**	2.6	(0.7–17.8)	1	(9)	33.3^b^,c	(0.8–84.2)	25	(83)	1.7	(0.6–17.2)	1	(3)
**CE2**	3.2	(1.0–13.1)	1	(9)	35.4^b^,c	(0.9–83.4)	24	(80)	2.5	(1.0–7.6)	1	(3)
E4	1.5	(0.4–6.0)	2	(18)	12.4^d^,c	(0.6–77.9)	13	(43)	1.0	(0.4–1.8)	0	(0)
E5	4.3	(0.6–16.5)	2	(18)	1.5^d^	(0.8–5.8)	1	(3)	1.1	(0.3–4.4)	1	(3)
**E6**	4.2	(0.9–9.6)	1	(9)	11.4^e^,c	(1.2–38.2)	15	(50)	2.9	(0.5–10.6)	1	(3)
**E7**	4.9	(0.8–16.6)	2	(18)	55.2^e^,c	(1.3–207.7)	19	(63)	1.4	(0.4–3.5)	0	(0)
L1	4.8	(0.8–17.2)	1	(9)	7.9^d^	(1.0–39.8)	7	(23)	3.3	(0.5–11.1)	1	(3)
L2	4.4	(1.1–21.3)	1	(9)	3.5^d^	(1.3–10.9)	2	(7)	2.3	(0.4–12.7)	1	(3)

Abbreviations: Ab=antibody; HPV=human papillomavirus; MFI=median fluorescence intensity.

a MFI ratio of HPV-GST antigen/p21-GST.

b Compared with partners, *P*<0.001.

c Compared with controls, *P*<0.001 (bold).

d Compared with partners, 0.001⩽*P*⩽0.05 unpaired Wilcoxon's test.

e Compared with controls, 0.001⩽*P*⩽0.05.
