# Supplementary material for: Serum antibodies to the HPV16 proteome as biomarkers for head and neck cancer
Source: Br J Cancer. 2011 Jun 7;104(12):1896–905. doi: 10.1038/bjc.2011.171 (PMC3111202; doi:10.1038/bjc.2011.171)
Supplement: Supplementary Tables 1 and 2 [file bjc2011171x1.doc]

Supplemental Table 1. HPV Primer design

| Type | Gene | 5’ primer | 3’ primer |
| --- | --- | --- | --- |
| HPV16 | E1 | AAAGCAGGCTCCACCATGGCTGATCCTGCAGGT | ACAAGAAAGCTGGGTCCAATAATGTGTTAGTATT |
|  | E1 | Inside: CAAAGGCAGCAATGTTAGCA |  |
|  | E2 | AAAGCAGGCTCCACCATGGAGACTCTTTGCCAA | ACAAGAAAGCTGGGTCCAATATAGACATAAATCC |
|  | NE2 | AAAGCAGGCTCCACCATGGAGACTCTTTGCCAA | ACAAGAAAGCTGGGTCCAATACTTTATTTTTACTATATTTTTC |
|  | CE2 | AAAGCAGGCTCCACCATGTGGGAAGTTCATGCGGGTGGTCAG | ACAAGAAAGCTGGGTCCAATATAGACATAAATCC |
|  | E4 | AAAGCAGGCTCCACCATGTATTATGTCCTACATCTG | ACAAGAAAGCTGGGTCCAATGGGTGTAGTGTTAC |
|  | E5 | AAAGCAGGCTCCACCATGACAAATCTTGATACTGC | ACAAGAAAGCTGGGTCCAATGTAATTAAAAAGCG |
|  | E6 | AAAGCAGGCTCCACCATGCACCAAAAGAGAACT | ACAAGAAAGCTGGGTCCAACAGCTGGGTTTCTCT |
|  | E7 | AAAGCAGGCTCCACCATGCATGGAGATACACCT | ACAAGAAAGCTGGGTCCAATGGTTTCTGAGAACA |
|  | L1 | AAAGCAGGCTCCACCATGCAGGTGACTTTTATT | Inside: GCAACATTGGTACATGGGGATCCTTTGCCCCAGTGTTCC |
|  | L1 | Inside:GGAACACTGGGGCAAAGGATCCCCATGTACCAATGTTGC | ACAAGAAAGCTGGGTCCAACAGCTTACGTTTTTT |
|  | L2 | AAAGCAGGCTCCACCATGCGACACAAACGTTCT | ACAAGAAAGCTGGGTCCAAGGCAGCCAAAGAGAC |
| HPV18 | E7 | AAAGCAGGCTCCACCATGCATGGACCTAAGGC | ACAAGAAAGCTGGGTCCAACTGCTGGGATGCACACCAC |
| Att | | GGGGACAAGTTTGTACAAAAAAGCAGGCTCC | GGGGACCACTTTGTACAAGAAAGCTGGGTC |

Supplementary Table 2. HPV16 Assay reproducibility

| **Gene product** | **Intra-assay** | **Inter-assay** |
| --- | --- | --- |
| E1 | 0.73-15.44 | 15.38 |
| E2 | 1.63-11.30 | 7.64 |
| E4 | 2.31-17.94 | 18.24 |
| E5 | 3.18-10.76 | 8.38 |
| E6 | 1.85-18.60 | 21.28 |
| E7 | 0.19-22.43 | 16.21 |
| L1 | 3.01-12.95 | 9.91 |
| L2 | 3.13-10.94 | 12.42 |
